# Physical violence and health-related quality of life: Danish cross-sectional analyses

**DOI:** 10.1186/1477-7525-10-113

**Published:** 2012-09-17

**Authors:** Jan Sørensen, Marie Kruse, Claire Gudex, Karin Helweg-Larsen, Henrik Brønnum-Hansen

**Affiliations:** 1 Centre for Applied Health Services Research and Technology Assessment, University of Southern Denmark, J.B. Winsløws Vej 9B, 5000, Odense, Denmark; 2 National Institute of Public Health, University of Southern Denmark, Øster Farimagsgade 5A, 1352, Copenhagen, Denmark; 3 Danish Institute of Health Services Research, Dampfærgevej 27-29, 2100, Copenhagen, Denmark; 4 Endocrinology Research Unit, University of Southern Denmark, Winsløwparken 19, 3rd floor, 5000, Odense, Denmark; 5 Department of Public Health, University of Copenhagen, Øster Farimagsgade 5, 1014, Copenhagen, Denmark

**Keywords:** Violence, Health-related quality of life, EQ-5D, SF-6D, General population, Denmark

## Abstract

**Background:**

The aim of this study was to evaluate the association between experienced physical violence and health-related quality of life (HRQoL) by comparing self-reported health status for individuals with and without experience of physical violence. Our hypothesis was that individuals exposed to violence would experience worse HRQoL than non-exposed individuals. We tested whether men and women and different age groups experience similar reductions in HRQoL, and the extent to which such differences might be associated with social circumstances and lifestyle conditions. Finally, we explored the HRQoL consequences of exposure to violence in a longer time perspective.

**Methods:**

We used data from self-completed questionnaires in two Danish nationally representative, cross-sectional health interview surveys. Exposure to violence was indicated through specific survey questions (Straus’ conflict tactics scale) enquiring about different types of violence during the last 12 months. Health status of respondents was elicited by the EQ-5D and SF-36 questionnaires. The health status profiles were converted to health score indexes using the Danish algorithm for EQ-5D and the revised Brazier algorithm for SF-6D. Differences in score indexes between the exposed and non-exposed individuals were explored separately for men and women using ordinary least square regression with four age categories as explanatory variables.

**Results:**

In the 2000 and 2005 surveys, respectively, 4.9% and 5.7% of respondents indicated that they had been exposed to physical violence within the last 12 months. Exposure to violence was more prevalent in the younger age groups and more prevalent for men than women. Respondents exposed to violence had lower score indexes on both the EQ-5D and the SF-6D compared with the non-exposed. Respondents who reported exposure to violence in both 2000 and 2005 reported lower HRQoL than individuals who only reported exposure in one of the surveys.

**Conclusions:**

The results of this study provide evidence for an association between exposure to physical violence and reduction in health-related quality of life.

## Background

The World Health Organisation recognizes violence as a public health problem, and violence against women is considered a priority health issue [1]. Violence may have both short- and long-term negative health consequences for survivors even after the abuse has ended. These effects can manifest as poor health status, poor quality of life and high use of health care services [2,3]. Violence during pregnancy, for example, has been associated with increased risk of low birth weight and perinatal foetal death [2,4].

Consequences on health-related quality of life (HRQoL) of violence may be measured using condition-specific or generic HRQoL instruments. Generic instruments are advantageous in that they allow comparison of the impact of violence on HRQoL with the impact of other conditions or illnesses. The Short Form–36 (SF-36) is an example of such a generic instrument [5]. Some generic HRQoL instruments provide a preference-based score for various health states (a numerical score that reflects the preferences of the general population regarding a health state compared to other health states). The EQ-5D instrument [6] and the SF-6D derived from the longer SF-36 instrument [7] are examples of such generic preference-based HRQoL measures.

We searched the research literature to identify studies that have assessed the consequences of violence on generic HRQoL. We identified relatively few studies that analyzed the consequences of exposure to violence on HRQoL using generic measures and no studies that used preference-based generic measures. These studies differ in terms of target group (women, men or both), type and definitions of violence (intimate partner violence, domestic violence including other family members) and study design.

The SF-36 was used in several studies. In a small Norwegian study where 87 women were interviewed about violence and HRQoL [8], women who had experienced violence had significantly lower scores on all SF-36 domains and especially for mental health and social functioning. Women who in telephone interviews reported recent violence had significantly poorer SF-36 social functioning due to physical or emotional problems [9-11]. Men with experience of intimate partner violence had lower SF-36 mental health scores than men without such experience, though no effect was found on SF-36 social or physical functioning; the effect on mental health was more marked for men aged 55 years and over [12]. Two studies that included both men and women used the shorter version SF-12 [13,14]; significantly lower SF-12 physical and mental health scores were associated with exposure to both physical assault and psychological abuse after adjustment for gender, danger assessment score and self-help score.

The Danish National Health Interview Surveys are large population-based representative surveys that are undertaken every five to six years [15]. The surveys have focused on a variety of health issues including respondents’ experience of physical violence [16,17] . We have earlier used the survey data to assess the costs associated with violence [18]. However, the surveys have also included HRQoL assessment using the EQ-5D and SF-36 instruments and these data have not previously been explored in relation to exposure to violence.

The aim of the current study was to evaluate the association between exposure to physical violence and health-related quality of life (HRQoL) by comparing self-reported generic preference-based HRQoL index scores for individuals with and without experience of violence.

Our hypothesis was that individuals exposed to violence would experience worse HRQoL than non-exposed individuals. We wanted to investigate whether there was a gender difference in the health impact of violence, and the extent to which the reported health impact might be associated with social and other lifestyle conditions. Finally, we aimed to explore HRQoL consequences of exposure to physical violence over a longer time perspective.

## Materials and methods

### Subjects

This study used data from the 2000 and 2005 National Health Interview Surveys (NHIS) [15]. The 2000 survey comprised three sub-samples: i) a nationally representative sample of approximately 6,000 individuals aged 16 years and above who were randomly selected from the Centralized Civil Register, ii) a follow-up sample of approximately 6,000 individuals from the entire (randomly selected) 1994 NHIS sample, and iii) a randomly selected supplementary sample to achieve approximately 1,000 individuals from each of the 15 Danish counties (except in Bornholm County, where 600 completed interviews were considered to be sufficient). The total 2000 NHIS sample thus comprised 22,484 individuals aged 16 years and above. These individuals were contacted by professional interviewers at the Danish National Centre for Social Research for a face-to-face interview that included questions on health, disability and health behaviour, as well as general questions regarding education, income and labour market status [19]. One-quarter of the sample either refused to be interviewed (22.4%) or was unable to participate for other reasons e.g. illness (3.4%). The 16,690 (74%) individuals who were interviewed were subsequently asked to complete the EQ-5D and the SF-36 as well as questions about physical violence. A total of 10,563 individuals (63% of the interviewed sample and 47% of the original NHIS sample) completed the supplementary questionnaires on violence, 98% also completed the EQ-5D and 83% completed the SF-36 (Table 1).

**Table 1:** Inclusion of participants from the Danish National Health Interview Surveys in 2000 and 2005

	**2000**		**2005**	
**Sampled**	**n = 22,484**		**n = 21,832**	
Responses to interveiw	16,690	74%	14,566	67%
Valid responses about violence	10,563	63%	5,202	72%*
-with valid EQ-5D responses	10,355	98%	-	
-with valid SF-6D responses	8,774	83%	4,835	93%
Responses to violence questions**	n = 10,563		n = 5,202	
Pushed, shaken	407	3.9%	168	3.2%
Kicked/hit	184	1.7%	67	1.3%
Thrown (e.g. against furniture, down stairs)	44	0.4%	30	0.6%
Strangulation/ weapon	30	0.3%	23	0.4%
Other	74	0.7%	88	1.7%

^*^Only half of the interviewed respondents were asked to complete questionnaire about violence.

^**^Respondents could record exposure to more than one type of violence but these data show the most severe violence reported.

The 2005 survey comprised two sub-samples: i) all those invited to the follow-up sample in 2000 if they were still alive and living in Denmark in 2005, and ii) a supplementary sample to ensure 3000 completed interviews in each of the five Danish regions. The total sample size was 21,832 individuals, of whom 14,566 (67%) participated in a face-to-face interview where half were asked to self-complete the SF-36 and a set of questions on physical and sexual violence. A total of 5,202 individuals (72% of those invited to complete the questionnaire) completed these supplementary questionnaires on violence and 93% also completed the SF-36 (Table 1).

### Exposure to violence

Exposure to violence was assessed by the use of specific questions [14]. In the 2000 survey, the question was phrased “Have you as an adult ever been subjected to one or more of the following forms of violence?”, followed by five categories of violence to which the respondents could indicate “Yes, within the past 12 months”, “Yes, previously” or “No”. The five categories were: “Pushed, shaken or stuck lightly”, “Kicked, struck with a fist or object”, “Thrown against furniture, into walls, down stairs or similar”, “Strangulation attempt, attack with a knife or firearm” and “Other types of violence”. The latter category could be described more specifically in an open-ended question. Respondents were also asked in a separate question whether they had ever been exposed to frightening threats of violence.

The wording in the 2005 survey was slightly different and focused only on the last 12 months: “Have you within the last year been exposed to one or more of the following forms of physical and sexual abuse?”. The five categories of physical abuse included “Threats of physical damage”, “Kicked, scratched, shaken, hit by flat hand or similar”, “Kicked, hit by fist or object”, “Thrown against furniture, into walls, down stairs or similar” and “Strangulation attempt, attack with a knife or firearm”, where the response categories were “Yes” or “No”. Respondents who answered “Yes” to any type of violence were asked to state the number of times they had been abused.

As our focus was on physical violence, data on exposure to threat of violence and exposure to sexual violence were not included in the analysis.

Due to the low number of responses in some of the categories of physical violence (Table 1), especially when stratified by age group, individuals exposed to physical violence during the past 12 months were defined as respondents who replied affirmatively to any one of the types of physical abuse (i.e. a dichotomous yes/no variable). The reported analyses thus do not distinguish between different types of physical abuse.

### HRQoL measures

The EQ-5D consists of five dimensions (mobility, self-care, usual activities, pain/discomfort and anxiety/depression), each with three levels (no problems, some problems, extreme/severe problems) [6,20]. These dimensions and levels combine into 243 different health states. A single index score was assigned to each health state using the Danish EQ-5D valuation algorithm [21], that was developed from regression modelling of preference data elicited from a representative sample (n = 1,332) of the Danish general population using the time trade-off valuation method. The index scores range from 1 (no problems on any EQ-5D dimension) to the lowest score of −0.624 (severe problems on all five EQ-5D dimensions).

The SF-6D is derived from responses to 11 questions in the SF-36 questionnaire. It covers six dimensions (physical functioning, role limitations, social functioning, pain, mental health and vitality), each with 4–6 levels. A single index score was assigned to each health state using the revised Brazier SF-6D valuation algorithm [22,23], that was developed from preference data elicited from a representative sample (n = 611) of the UK general population using the standard gamble valuation method. The index scores range from 1 (full health) to the lowest score of 0.296 (worst SF-6D health state).

### Social characteristics

Level of schooling and highest completed professional education (based on the UNESCO international standard classification of education) were combined into a variable of education duration: less than 10 years, 10 years completed, 11–12 years completed, 13–14 years completed, 15+ years completed and ‘other’ including still in education, foreign education and missing data [24] (Appendix A, p. 536–7). Marital status was combined with data on civil status and cohabitation into four categories: married, cohabiting, single and unknown. Living with children (yes/no) was based on a question asking how many children were living in the household. Weekly alcohol intake was based on number of standard units imbibed during the last week, and was categorized as 14 or less, 15–21 and 22 or more units per week. Use of medication (yes/no) was based on self-reported use of medicines during the previous two weeks.

### Statistical analyses

Data from the two samples (2000 and 2005) were analyzed separately and stratified by gender. Age was categorized in broad groups to maintain statistical power (16–29 years, 30–39 years, 40–49 years, 50–59 years and 60+ years). Chi-squared tests were used to compare the distribution of categorical data, and unpaired t-tests were used to test mean differences in the EQ-5D and SF-6D score indexes.

Ordinary least squares regression models were used to test the hypotheses concerning HRQoL differences. Separate models were estimated for each HRQoL instrument, where the HRQoL score index was used as the dependent variable. In *Model 1*, the explanatory variables were the four age categories (reference = 16–29 year-olds) and a dummy variable for exposure to violence (=1). The estimated parameters for age categories represent the average change in HRQoL from the reference group; negative estimates were expected as HRQoL typically deteriorates with increasing age. The estimate for violence exposure represents the average difference in score index for those who are exposed to violence; a negative estimate was expected as individuals exposed to physical violence were assumed to have reduced HRQoL.

In *Model 2* we investigated whether exposure to physical violence had a greater effect on older age groups and introduced a set of interaction variables between exposure and age category. The parameters of these interaction variables were interpreted as the marginal change in HRQoL for individuals in the particular age group, who had been exposed to violence.

In *Model 3* social and lifestyle characteristics were introduced as explanatory variables. As some of the HRQoL differences between persons exposed and non-exposed to violence might be related to social factors, we expected the HRQoL loss associated with violence exposure to reduce but remain negative.

#### Longitudinal analysis

Respondents who had responded to the questions on physical violence in both the 2000 and the 2005 survey could be identified by their unique person identification number. These respondents were categorized as: a) reporting exposure to violence in either 2000 or 2005, b) reporting exposure to violence only in 2000 or c) only in 2005, or d) reporting exposure to violence in both 2000 and 2005. The change in SF-6D index score between the 2000 and 2005 surveys was calculated. To explore the consequences of long term physical violence exposure, a random effect regression model was fitted for both genders (to maintain statistical power), with the index score as the dependent variable and the categories of violence exposure (three binary variables), gender and age as explanatory variables. All other things being equal, we expected the HRQoL of non-exposed individuals to stay the same or be slightly reduced due to age-related health deterioration over the five years. Individuals exposed to violence in 2000 but not exposed in 2005 were expected to show an improvement in HRQoL, while those with new exposure in 2005 were expected to show deterioration in HRQoL. Individuals exposed to violence in both years were expected to have deterioration of HRQoL over time. As a supplementary analysis, the score index in 2000 was included as an explanatory variable in order to improve the fit of the model.

All statistical analyses were undertaken using Stata MP – parallel edition, version 11 at Statistics Denmark’s research server. P-values < 0.05 were considered significant.

## Results

In the 2000 and 2005 survey, respectively, 520 of 10,563 respondents (4.9%) and 296 of 5,202 (5.7%) respondents indicated that they had been exposed to physical violence (Table 2). In 2000, 6.2% of men (out of 5041) and 3.8% of women (out of 5522) had been exposed to violence. In 2005 it was 6.4% (of 2407 men) and 5.0% (of 2795 women). Exposure to violence was also more prevalent among those aged 16–29 years. Chi-square tests also indicated greater exposure to violence amongst those with least education, single respondents (only in 2000), with higher weekly alcohol intake and not using medication.

**Table 2:** Comparison between respondents with and without exposure to physical violence in the last 12 months

	**2000**					**2005**				
**Violence**	**Exposed**		**Unexposed**		**chi**^**2**^	**Exposed**		**Unexposed**		**chi**^**2**^
All	520		10,043			296		4,906		
Gender										
Males	312	60%	4,729	47%	p < 0.01	155	52%	2,252	46%	p < 0.01
Females	208	40%	5,314	53%		141	48%	2,654	54%	
Age										
16-29 yrs	313	60%	1,838	18%	p < 0.01	143	48%	716	15%	p < 0.01
30-39 yrs	98	19%	1,808	18%		68	23%	857	17%	
40-49 yrs	58	11%	2,014	20%		43	15%	1,012	21%	
50-59 yrs	35	7%	2,009	20%		29	10%	1,015	21%	
60+ yrs	16	3%	2,374	24%		13	4%	1,306	27%	
Education										
Less than 10 years	64	12%	1,734	17%	p < 0.01	19	6%	578	12%	p < 0.01
10 years	61	12%	521	5%		28	9%	236	5%	
11-12 years	110	21%	2,457	24%		53	18%	1,067	22%	
13-14 years	155	30%	3,091	31%		100	34%	1,669	34%	
15+ years	89	17%	2,065	21%		74	25%	1,243	25%	
Currently under education	34	7%	108	1%		20	7%	63	1%	
Other including unknown	7	1%	67	1%		2	1%	50	1%	
Marital status										
Married	123	24%	5,827	58%	p < 0.01	76	26%	3,005	61%	p = 0.10
Cohabiting	101	19%	1,475	15%		63	21%	705	14%	
Single	283	54%	2,676	27%		157	53%	1,1795	24%	
Unknown	13	3%	65	1%		-	0%	1	0%	
Living with children	179	34%	3,073	31%	p = 0.07	104	35%	1,499	31%	
Weekly alcohol intake										
14 or less	368	71%	8,310	83%	p < 0.01	218	74%	3,912	80%	p = 0.04
15-21 units	74	14%	967	10%		39	13%	491	10%	
22+ units	78	15%	766	8%		39	13%	503	10%	
User of prescription medication	111	21%	3,341	33%	p < 0.01	90	30%	1,864	38%	p = 0.01
EQ-5D index score (mean, sd)	0.88 (0.15)		0.88 (0.16)		p = 0.94^a^	-		-		
SF-6D index score (mean, sd)	0.79 (0.11)		0.81 (0.12)		p = 0.01^a^	0.78 (0.11)		0.82 (0.11)		p < 0.01^a^

^a^ indicate *t*-test.

The unadjusted mean EQ-5D score index in 2000 was 0.88 with no statistically significant difference between respondents exposed and unexposed to violence. The mean SF-6D score index was lower than the EQ-5D score index. Exposure to violence was significantly associated with lower mean unadjusted SF-6D index score in both the 2000 and the 2005 survey.

The results of the gender-stratified OLS regressions with score index as dependent variable and exposure to physical violence and age categories as explanatory variables (*Model 1*) are shown in Table 3. Most of the estimated parameters were statistically significant (p < 0.01) with a few exceptions for SF-6D index score in the 2005 sample. The HRQoL of both men and women exposed to violence was statistically lower than non-exposed individuals. The estimated parameters for the age categories had the expected signs and supported the assumption of lower HRQoL in older age groups (deterioration with age). Women had a lower HRQoL score than men but the effect of exposure to violence was similar for both genders in terms of reduction of HRQoL.

**Table 3:** OLS regression of health-related quality of life index scores and exposure to physical violence, by age and stratified by gender

	**EQ-5D**		**SF-6D**		**SF-6D**	
	**2000**		**2000**		**2005**	
	**est**	**95% CI**	**est**	**95% CI**	**est**	**95% CI**
*Men*						
Violence	−0.032	(−0.049;-0.015)	−0.036	(−0.049;-0.022)	−0.050	(−0.068;-0.031)
30-39 yrs	−0.018	(−0.031;-0.004)	−0.013	(−0.027; 0.007)	−0.001	(−0.014;-0.015)
40-49 yrs	−0.040	(−0.053;-0.027)	−0.018	(−0.024;-0.003)	−0.017	(−0.032;-0.002)
50-59 yrs	−0.062	(−0.075;-0.049)	−0.032	(−0.042;-0.022)	−0.038	(−0.053;-0.023)
60+ yrs	−0.091	(−0.104;-0.079)	−0.061	(−0.071;-0.051)	−0.043	(−0.058;-0.029)
constant	0.947	(0.938;0.957)	0.854	(0.846;0.862)	0.857	(0.846;0.869)
no. observations	4953		4735		2273	
adjusted R^2^	0.05		0.04		0.03	
*Women*						
Violence	−0.041	(−0.065;-0.018)	−0.056	(−0.073;-0.039)	−0.059	(−0.08;-0.038)
30-39 yrs	−0.014	(−0.028;0)	−0.001	(−0.009;0.011)	0.004	(−0.011;-0.018)
40-49 yrs	−0.042	(−0.056;-0.028)	−0.010	(−0.021;-0.001)	−0.005	(−0.019;0.01)
50-59 yrs	−0.069	(−0.083;-0.054)	−0.018	(−0.029;-0.008)	−0.009	(−0.024;0.006)
60+ yrs	−0.116	(−0.13;-0.102)	−0.061	(−0.072;-0.051)	−0.24	(−0.038;-0.009)
constant	0.912	(0.909; 0.929)	0.815	(0.808;0.822)	0.082	(0.805;0.827)
no. observations	5402		5036		2562	
adjusted R^2^	0.06		0.04		0.02	

Age 16–29 years used as reference.

est: parameter estimate.

95% CI: 95% confidence interval.

The results for *Model 2*, which included interaction variables between exposure to physical violence and age category, showed clear tendencies for both men and women where exposure to violence and older age was associated with lower HRQoL. All estimated parameters were negative. However, only a few of the parameters for interaction variables reached statistical significance (men aged 50–59 and exposed to violence, n = 33). The deteriorations in HRQoL for older age categories remained statistically significant (data not shown).

Table 4 shows the estimations resulting from the models that included social factors as explanatory variables (*Model 3*). The model fit improved as expected, with R-squared ranging from 0.11 to 0.16. The estimated differences in HRQoL were reduced but remained statistically significant in all models.

**Table 4:** OLS regression of health-related quality of life index scores and exposure to physical violence adjusted for age categories, alcohol consumption, civil status, living with children, education and current use of medicine; stratified by gender

	**EQ-5D**		**SF-6D**		**SF-6D**	
	**2000**		**2000**		**2005**	
	**est.**	**95%-CI**	**est.**	**95%-CI**	**est.**	**95%-CI**
*Men*						
Violence	−0.024	(−0.041;-0.008)	−0.028	(−0.041;-0.015)	−0.035	(−0.018;-0.051)
Adj. R^2^	0.14	n = 4953	0.12	n = 4735	0.11	n = 2273
*Women*						
Violence	−0.038	(−0.080;-0.015)	−0.052	(−0.068;-0.035)	−0.045	(−0.027;-0.063)
Adj. R^2^	0.16	n = 5402	0.11	n = 5036	0.11	n = 2562

est.: parameter estimate.

95% CI: 95% confidence interval.

Inclusion of gender into the regression model showed an expected difference in score index between men and women of 0.03 on both EQ-5D and SF-6D (2000 and 2005). However, the interactions between gender and age categories were insignificant in all three samples, suggesting an age-independent difference in HRQoL for men and women. The inclusion of interaction between violence and gender provided small and insignificant parameter estimates, suggesting that the difference in HRQoL between those exposed and unexposed to physical violence is independent of gender (data not shown).

Analysis of long-term exposure to physical violence was undertaken using only the SF-6D index score, as EQ-5D data were not collected in the 2005 survey. Of the 1972 respondents (47% men) who participated in both the 2000 and the 2005 survey, 63 reported exposures to violence in 2000 but not in 2005; 78 reported exposure to violence in 2005 but not in 2000 and 20 reported exposure to violence in both 2000 and 2005. The remaining 1811 respondents (92%) did not report exposure to violence in either survey.

The non-exposed respondents had a small, insignificant fall in SF-6D index score (p = 0.46). The health index score reduced by −0.029 (p = 0.02) for those exposed to physical violence in 2000 but not in 2005. Respondents who had been exposed to violence in 2005 but not in 2000 showed no difference in HRQoL (p = 0.94), whereas respondents who reported exposure to violence in both 2000 and 2005 showed an insignificant reduction in HRQoL of 0.041 (p = 0.10). The model that included variables for social factors provided similar results, with a slightly larger difference for individuals exposed for violence in 2000 but not in 2005 and a smaller difference for respondents exposed for violence in both 2000 and 2005.

## Discussion

The main objective of this study was to assess the association between exposure to physical violence within the past 12 months and self-reported health status. The analysis was based on data from two nationally representative health interview surveys undertaken in 2000 and 2005.

The study results suggest that 5-6% of Danish adults have experienced physical violence during the last year. The numbers are slightly higher for 2005 than 2000, but are within the bounds of statistical uncertainty. More men than women reported physical violence: 6.2% and 6.4% of men in 2000 and 2005, respectively, compared to 3.8% and 5.0% of women. Young people were more likely than others to be exposed to violence: nearly one out of five men and one out of ten women aged 16–29 years had experienced violence during the last year, although experience of violence was also reported by older age groups. Exposure to violence was also more likely for individuals with less education, single people and those with a high weekly alcohol intake. These findings are similar to those found in [3,18].

Individuals who had experienced physical violence had lower HRQoL according to the EQ-5D and SF-6D instruments. After adjustment for age category and stratification for gender, the index score differences ranged from 0.032 to 0.058 depending on the instrument used and the survey year. Research into minimally important differences suggests that health states with less than 0.03 units difference on a generic utility-based index cannot be considered as different from one another [25]. The minimally important difference for the SF-6D has been suggested at 0.033 [26], while the minimally important difference for EQ-5D appears to be higher (0.06-0.08) [27,28].

With these minimally important differences in mind, there appeared to be only a small difference between men and women in the HRQOL consequences of violence, although women exposed to violence had a larger reduction in HRQOL than men. When interpreting such gender differences it is important also to consider that there is a gender difference in the general population in HRQoL where men score higher than women [29,30]. The gender difference might thus be larger than suggested by the data presented.

Although there was some evidence of older individuals experiencing more severe consequences in terms of reduced HRQOL than younger individuals, this finding was not statistically significant. Lower HRQoL can also be related to social and lifestyle factors [31]. When the analysis took such variables into account, the difference in HRQoL score between the exposed and non-exposed individuals reduced but remained statistically significant in nearly all analyses.

Individuals who were exposed to physical violence in both 2000 and 2005 showed a reduction in HRQoL score over time of 0.04 (p = 0.10), though this result was not statistically significant – possibly due to the low statistical power as only 20 individuals (1.0%) reported exposure to violence in both surveys.

While the main finding of this study is a consistent association between exposure to violence and lower HRQoL, the causality of this relationship is not clear. A number of the risk factors for violence exposure, such as educational level and marital status, have themselves an impact on HRQoL. We found no strong confirmation that continued exposure to violence reduces HRQoL even further, although there was a non-significant trend towards this.

These results using generic health measures fit well with results from other studies reporting effects on physical and psychological health measured using non preference-based instruments. Thus, several studies report significant associations between current intimate partner violence and physical and/or psychological symptoms after adjustment for lifestyle factors [32,33]. An association has also been reported between exposure to physical or sexual violence and increased frequency of somatic symptoms and/or diseases, after controlling for sociodemographic factors [33,34].

Generic preference-based HRQOL measures are increasingly used to describe population health status and to assess outcome from health care interventions. The major advantage of such measures is the opportunity to compare scores across groups with very different health problems. Preference-based measures also allow interventions to be compared in terms of their social value.

### Strengths and limitations of the study

The main strength of the study is its basis in a large nationally representative population sample using paper-based questionnaires to assess exposure to violence. This approach may be less susceptible to underreporting of violence due to embarrassment and social stigma than for example data collection through personal interviews [31].

Our definition of exposure to physical violence was fairly general being based on yes/no responses to questions about specific types of violence included in national health surveys. We explored regression models that included the different subtypes of violence reported in the surveys and found that more severe types of violence tended to have greater impact on HRQoL, but these analyses relied on relatively few observations (data not reported).

Respondents in the 2000 survey also reported frequency of exposure to physical violence within the last year. However, inclusion of a frequency variable did not improve the fit of the regression models and the estimated parameter was not significant. It was unexpected that the application of more detailed description of violence exposure (type and frequency of violence) did not improve the regression models, as severity and frequency of violence would be expected to influence HRQoL. This finding may be due to a weak linear association between frequency of violence exposure and HRQoL deterioration.

Although the study population was sampled to be representative of the general population, the low response rate for parts of the data collection may be a concern. As the prevalence of exposure to violence was relatively low (5-6%) in the study population as a whole, the sample of exposed individuals was limited. The statistical power for subgroup analysis was especially weak in the longitudinal analysis, where only few of the participants reported violence exposure at both survey points.

### Difference between instruments

As in previous studies [35-37] the EQ-5D and SF-6D instruments did not generate directly comparable scores. Although they both provide preference-based index scores of HRQoL their preference scores are derived using different valuation and modelling methods, and in this case also preferences from two different countries. A direct comparison of the raw scores should thus be avoided, but the results give an indication of the degree to which violence can affect HRQoL. The completion rate for the SF-6D was lower (83%) than for EQ-5D (98%). However, the SF-6D appeared more sensitive than the EQ-5D to the adverse impacts of exposure to violence on health status. The EQ-5D also showed a ceiling effect, where 94% of the sample in the 2000 survey reported full health (a score of 1.0) on the EQ-5D, compared to only 3% on the SF-6D instrument. Finally, neither of the instruments generated index scores with a normal distribution, also shown by analysis of the residuals. Care should thus be taken when interpreting the statistical uncertainties of the estimates from linear regression, as they are based on an assumption of normally distributed residuals, although the large number of observations makes the modelling results more robust.

## Conclusion

This study found that respondents exposed to physical violence had lower score indexes on both the EQ-5D and the SF-6D compared with the non-exposed. Respondents who reported exposure to violence in both 2000 and 2005 reported lower HRQoL than individuals who only reported exposure in one of the surveys. The results of this study provide evidence for an association between exposure to physical violence and reduction in health-related quality of life.

## Competing interests

The authors declare that they have no competing interest.

## Authors’ contributions

This study was developed as part of a long-term collaboration between KHL, HBH, MK and JS. The first draft of the manuscript and the statistical analyses were devised by JS and discussed with KHL, HBH and MK. All authors contributed to the contents. CG revised the manuscript. All authors read and approved the final manuscript.
